# Cognitive Profiles in Parkinson's Disease and Their Relation to Dementia: A Data-Driven Approach

**DOI:** 10.1155/2012/910757

**Published:** 2012-10-18

**Authors:** Inga Liepelt-Scarfone, Susanne Gräber, Monika Fruhmann Berger, Anne Feseker, Gülsüm Baysal, Ilona Csoti, Jana Godau, Alexandra Gaenslen, Heiko Huber, Karin Srulijes, Kathrin Brockmann, Daniela Berg

**Affiliations:** ^1^Department of Neurodegeneration, Hertie Institute for Clinical Brain Research, University of Tuebingen, Hoppe-Seyler Str. 3, 72076 Tuebingen, Germany; ^2^German Center of Neurodegenerative Diseases (DZNE), 72076 Tuebingen, Germany; ^3^Department of Neurology, Gertrudis Hospital, 35638 Leun-Biskirchen, Germany

## Abstract

Parkinson's disease is characterized by a substantial cognitive heterogeneity, which is apparent in different profiles and levels of severity. To date, a distinct clinical profile for patients with a potential risk of developing dementia still has to be identified. We introduce a data-driven approach to detect different cognitive profiles and stages. Comprehensive neuropsychological data sets from a cohort of 121 Parkinson's disease patients with and without dementia were explored by a factor analysis to characterize different cognitive domains. Based on the factor scores that represent individual performance in each domain, hierarchical cluster analyses determined whether subgroups of Parkinson's disease patients show varying cognitive profiles. A six-factor solution accounting for 65.2% of total variance fitted best to our data and revealed high internal consistencies (Cronbach's alpha coefficients >0.6). The cluster analyses suggested two independent patient clusters with different cognitive profiles. They differed only in severity of cognitive impairment and self-reported limitation of activities of daily living function but not in motor performance, disease duration, or dopaminergic medication. Based on a data-driven approach, divers cognitive profiles were identified, which separated early and more advanced stages of cognitive impairment in Parkinson's disease without dementia. Importantly, these profiles were independent of motor progression.

## 1. Introduction

Beyond the characteristic motor signs, a number of nonmotor symptoms including cognitive aspects are gaining increasing attention in Parkinson's disease (PD). Recent work revealed a substantial heterogeneity of cognitive impairment, which is apparent in both different profiles and different levels of severity ranging from slight and early cognitive changes up to the diagnosis of PD dementia (PDD) [1]. With the demand for an early, individualized, and better therapeutic treatment, the focus is now to identify the patients with a potentially higher risk of dementia [2]. Already a few cognitive alterations may enhance the risk of PDD. However, it needs to be noted that exactly the same cognitive alterations can also be found in PD patients who did not develop dementia [3, 4]. On subtest level, some studies suggest that early executive dysfunction is predictive of the conversion to dementia [2, 3], while others argue for a crucial role of impaired visuospatial and language abilities [4]. Thus, a distinct clinical profile for patients with a potential risk of developing PDD still has to be identified [1].

Most authors define the stage of mild cognitive impairment in PD (PD-MCI) [5] and the involvement of different cognitive domains by a predominately theoretical [6, 7] rather than a data-driven, quantitative approach. Some data-driven studies on different subtypes of PD found a poor test performance in varying neuropsychological tasks, suggesting that these help to diagnose PDD [8, 9]. Recently, a cluster analysis on a small cohort of PD patients without dementia revealed differences in severity of cognitive deterioration but not in cognitive phenotypes [10]. We here propose an approach to identify cognitive profiles based on performance differences within quantitatively determined domains using standardized factor scores. These standard values can be used to compare the mean performance of each single PD patient investigated here to the mean performance of the present, total PD cohort. Our study thus was designed by (i) a data-driven identification of different cognitive domains in a large cohort of both PD patients with and without dementia and (ii) a data-based subdivision of PD patients according to exactly these standardized domain scores to characterize subgroups with divers cognitive profiles and potentially different levels of cognitive impairment.

## 2. Materials and Methods

### 2.1. Patients

We investigated 121 patients with idiopathic PD according to the UK Brain Bank criteria [11] admitted to the outpatient clinic of the Department of Neurodegenerative Diseases University of Tuebingen and the Gertrudis Clinic Leun-Biskirchen Germany. All patients received their usual, optimized medication and were able to complete all cognitive tasks (Tables 1 and 2 provide all relevant details).

Inclusion criteria were: age ≥ 50 years, onset of dementia >2 years from PD diagnosis, adequate or corrected hearing/visual abilities, and German as first language. Exclusion criteria were: other neurological diseases affecting the central nervous system, prior surgery for PD, medication interfering with cognition (i.e., hypnotics or tranquilizers), or a minimental state examination [12] score < 18 (testing not feasible). Patients with identified gene mutations and those reporting more than 2 first or second degree relatives with a definitive diagnosis of PD [7] were excluded to avoid monogenetic subgroups in which cognition can be specifically altered [13]. Most patients identified their spouse as caregiver (72.7%); the others indicated an adult child (12.4%), other family members (8.3%), or nonfamily members (6.6%). The study was approved by the local ethical committee. All patients and caregivers gave written informed consent.

### 2.2. Neuropsychological Assessment

All examinations and expert evaluations were carried out within a week. Each patient underwent a comprehensive test battery according to the recommendations of the Movement Disorder Society (MDS) Task Force [7] comprising the following tests (see also supplemented Table 1 in Supplementary material available online in doi:10.1155/2012/910757): the Tower of London (TL-D, conceptualization) [14], the Trail Making Test parts A and B (TMT-A, TMT-B; psychomotor speed, set shifting) [15], the digit span (DS forward and backward, working memory capacity), and the figure test (FT, nonverbal memory, set maintenance) from the Nuernberger Alters Inventory a battery to assess mild to advanced cognitive impairments (NAI) [16], as well as 8 subtests, that is, word-list memory (WL), including the number of false positive words (WL-I), word-list recall (WL delay), word-list recognition (WL-R, all verbal memory), the Boston naming test (BNT, language), verbal fluency (VF, animal naming, executive function), as well as the copy task (praxis) and its delayed recall (praxis delay, both visuospatial abilities) from the German version of the Consortium to Establish a Registry on Alzheimer's Disease (CERAD) [15]. Further, we applied the logical memory tasks (LogI and LogII) of the Wechsler Memory Scale-Revised (WMS-R, verbal memory) [17], the object decision part (OD) of the Visual Object and Space Perception battery (VOSP, visuospatial abilities) [18], the Berlin Apraxia Test (BAXT, ideomotor apraxia) [19], and two computerized reaction time tasks (Alertness, Go-Nogo; Test of Attentional Performance, TAP) [20], the first providing a specific measure of the ability to respond to a critical stimulus following an auditory cue (phasic alertness). Analyses are based on standard norms (percentile rank scores) of healthy German control subjects as published in the manuals. Data are corrected either for age (NAI, WMS-R, VOSP) or for age and education (CERAD, TAP, TMT, TL-D).

*Diagnostic criteria for dementia* followed the recommendation of the MDS task force for probable PDD [7]. In detail, our criteria for PDD were (i) scores 1.5 SD (PR < 7) in at least one test below published group norms of healthy control subjects in at least 2 of the following cognitive domains: attention (as measured by Alertness, Go-Nogo), executive function (DS, TL-D, TMT-A, TMT-B, VF), visuospatial function (praxis, praxis delay, OD), memory (LogI, LogII, WL, WL-I, WL delay, WL-R, FT), or language ability (BNT), (ii) cognitive decline with insidious onset and slow progression reported by either the patients or their proxies, and (iii) impairment of nonmotor activities of daily living (ADL) as verified by a structured patient and/or caregiver interview on the perception of cognitively influenced ADL function in the domestic environment.

### 2.3. Motor Performance, Behavioral Disturbances, and ADL

Clinical assessment included the Hoehn and Yahr stage, the unified Parkinson disease rating scale part III (UPDRS-III) [21] for motor function and the neuropsychiatric inventory (NPI) for behavioral disturbances (e.g., hallucinations) [22]. The Parkinson's Disease Questionnaire (PDQ-39) [23] and the Beck's Depression Inventory (BDI) [24] provided self-rating scales of health related quality of life and mood. Further, we calculated age-corrected standard scores of the patients' ADL function using (i) a self-rating questionnaire (Nuernberger-Alters-Alltagsaktivitaeten-Skala, NAA) and (ii) its corresponding scale for proxies (Nuernberger-Alters-Beobachtungsskala, NAB) [16].

Full drug history includes the total daily dose of levodopa only and the total daily dose of all dopaminomimetics, which was calculated as levodopa equivalent daily dose (LEDD) according to published conversion rates (see legends of Tables 1 and 2, and [25–27]).

### 2.4. Data Analyses

#### 2.4.1. Identification of Cognitive Domains: Exploratory Factor Analysis (EFA)

First, we performed an EFA on all neuropsychological data to identify cognitive domains in the PD group. The factor matrix was optimized with oblique rotation, because factors were expected to be correlated [28]. Variables with a factor loading >0.5 or <−0.5 were considered as core variables for a given factor [29]. The Kaiser's criterion (eigenvalue > 1) and the corresponding scree plot results were used to determine the number of factors to be retained. Internal consistency was verified by Cronbach's alpha (*α*) coefficient, which was required to be higher than *α* > 0.6 for each factor to indicate a sufficient internal consistency structure [6].

#### 2.4.2. Identification of Characteristic Profiles in the PD Cohort: Hierarchical Cluster Analysis

Based on the factor loadings from the EFA, individual factor scores were calculated by the Anderson-Rubin algorithm, which produces factor scores that are uncorrelated and standardized with a mean of zero and a standard deviation of 1. A patient with a factor score of 0 (zero) thus shows average performance compared to the total PD cohort; a positive score indicates performance above, a negative score below the average of the whole patient group investigated here.

With these individual factor scores, we performed two separate hierarchical cluster analyses to identify patient subgroups with different cognitive profiles (Ward's-method). The first analysis was conducted on the total group of PD patients (*n* = 121), the second, for validation purposes, on all PD patients except those with dementia (*n* = 97). As PDD patients can be expected to suffer from more severe cognitive, motor, and behavioral impairment, we evaluated this specific influence on our study results by excluding them from the second HCA. Student's *t*- (age, disease duration, UPDRS-III motor score) or *χ*²-tests (gender, Hoehn & Yahr stage) were used for between-group comparisons. Analyses of covariance and Mantel-Haenszel statistics accounted for differences in demographics and disease severity. Because of the number of comparisons, the significance levels were set at *P* = 0.01 to optimize the trade-off between false positive protection (type 1) and sensitivity/power (type 2 error). All analyses were conducted using SPSS 17.0 (SPSS Inc, Chicago, III, USA).

## 3. Results

### 3.1. Patient Characteristics

Of the 121 patients, seventeen (14.0%) received L-dopa, 25 (20.7%) dopamine agonists, and one (0.8%) patient amantadine only. Both L-dopa and dopamine agonists were given to 78 (64.5%) patients, of whom 27 additionally received amantadine. Twenty-four patients (19.8%) of the total cohort had PDD; six of them were treated with cholinesterase inhibitors (see Table 1 for further details).

### 3.2. Cognitive Domains

Table 3 shows the result of the EFA and the internal consistency analysis. The EFA was verified by the Bartlett's test of sphericity (*χ* ^2^ = 872.7, *df* = 171, *P* < 0.001) and the Kaiser-Meyer-Olkin measures of sampling adequacy (MSA = 0.82). A six-factor solution accounting for 65.2% of total variance explained by the factors fits best to our data (see supplemented Table 1 for information on the rejected five-factor solution). The internal consistency structure of each factor was found to be at least moderately high (0.67 ≤ *α* ≤ 0.86). Factor 1 consisted of five neuropsychological tasks explaining 33.5% of total variance (*α* = 0.67). Like the TL-D [14], each test was mainly related to aspects of frontal lobe function. Factor 2 comprised four tasks on word-list memory and recall (*α* = 0.78, 8.2% of variance explained). Both Factor 3 (6.8% of variance explained, *α* = −0.85) and Factor 4 (5.9%, *α* = 0.86) consisted of two neuropsychological tasks on attention and episodic memory (5.7% of variance explained), respectively. Factor 5 consisted of tasks on praxis and visual perception (*α* = 0.70, 5.7% of variance explained). Factor 6 comprised three tests that are mainly used for assessing fluency and naming ability (5.2% of variance explained, *α* = 0.70). 

### 3.3. Cognitive Profiles in PD

**Figure 1:**
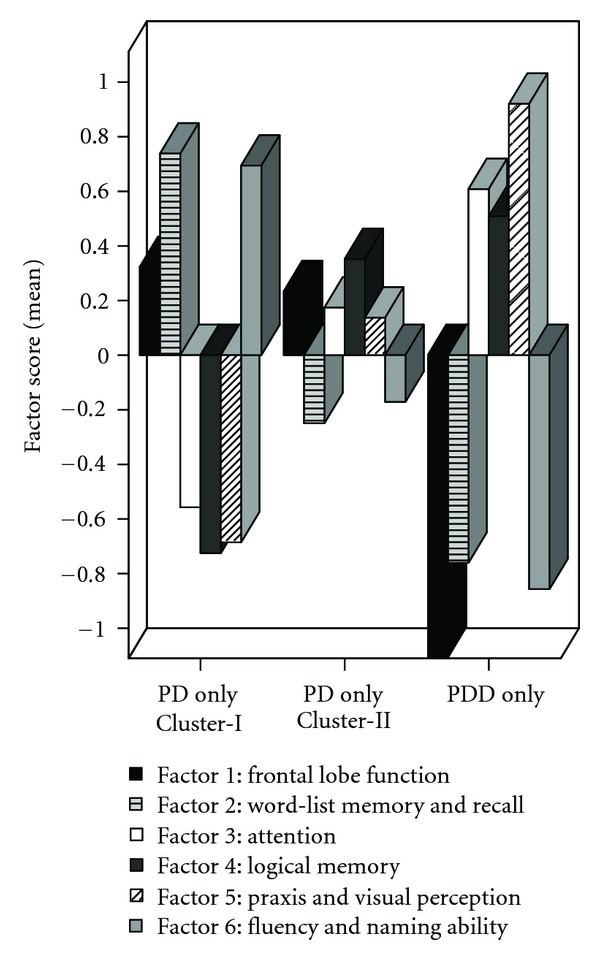
Mean group performance (mean factor scores) of PD patients without dementia clustered into two distinct groups (PD only, *n* = 43, Cluster-I versus PD only, *n* = 54, Cluster-II) as well as of the group of PD patients with dementia (PDD only, *n* = 24).

Table 1 refers to the hierarchical cluster analysis on the total group of PD patients (*n* = 121, incl. 24 PDD), Table 2 to the subsequent hierarchical cluster analysis on all PD patients except those with dementia (*n* = 97). Both analyses revealed two different, independent clusters regarding the degree of cognitive impairment within the domains defined by the EFA (see Figure 1). Our first hierarchical cluster analysis assigned all 24 patients with PDD to Cluster-II, that is, to the group with poorer neuropsychological test performances (see Table 1, PD and PDD). In contrast, all Cluster-I patients showed less cognitive impairment and, crucially had no dementia (“PD only”). The second hierarchical cluster analysis, carried out for validation purposes, replicated the grouping in 92.8% of all PD patients without dementia (*n* = 97, Table 2). Most important, no patient initially assigned to the more severely impaired Cluster-II, was regrouped in Cluster-I.

Our approach of identifying characteristic profiles within the PD cohort (i.e., an identification of those patients with poorer individual performances in the specific tests of the corresponding EFA domain compared to all other PD patients investigated here) revealed a clear-cut division of the six cognitive domains into two subgroups (all *P* values < 0.005). PD patients with lower factor scores of Factor 3 (attention *P* < 0.001), Factor 4 (logical memory *P* < 0.001), and Factor 5 (praxis and visual perception, *P* < 0.001) were grouped in Cluster-I. In contrast, Cluster-II patients showed lower factor scores in neuropsychological tasks assigned to Factor 1 (frontal lobe function, *P* < 0.005), Factor 2 (word-list memory and recall, *P* < 0.001), and Factor 6 (fluency and naming ability, *P* < 0.001). The analysis without PDD patients revealed comparable results except for frontal lobe functions (Figure 1).

### 3.4. Subgroup Comparison of Clinical Parameters in Patients with “PD Only”

To identify a cognitive profile in patients who had not developed dementia at the time of examination, we compared the clinical parameters of the two Clusters without PDD (Table 2). Cluster-II tended to be older (*P* < 0.02). As age is a risk factor for PDD [30], all *P* values were corrected for it.

No differences were found for motor disability, disease duration, and psychiatric symptoms. Compared to published group norms that are standardized on healthy control subjects (not to the present factor scores), Cluster-II patients showed overall lower performances in most neuropsychological tests. However, comparison on subgroup level without PDD failed significance, for example, for most executive tasks (see Table 2), suggesting that particularly PDD patients had led to significant differences in the first hierarchical cluster analysis because of marked difficulties in this domain.

Regarding the impact of ADL dysfunction on PD, it is notable that members of Cluster-II (without PDD) rated themselves as more impaired (*P* = 0.002). These self-impressions tended to be confirmed by their caregivers as well as by their reduced quality of life reports (PDQ-39, *P* = 0.03).

## 4. Discussion

We introduce a data-driven approach to identify different profiles and stages of cognitive impairment in PD. First, we determined cognitive domains based on a comprehensive neuropsychological test battery, and second we identified subgroups that differ with respect to their standardized individual performance in these domains. Whereas the first part has already been addressed to some extent [8–10, 31], the second part provides a first attempt to identify PD patients with a potentially higher PDD risk using PD related rather than healthy control norms. This method allows the differentiation within the group of those PD patients, who show a severe impairment in almost all cognitive tasks and who thus might have a potential risk of developing dementia. While the standard procedure (i.e., using healthy control standard norms) turned out insufficient to differentiate within this overall severely impaired patient group, our approach of using factor scores revealed varying cognitive profiles that differ with respect to both, severity and most affected cognitive functions.

### 4.1. Factor Analysis

Focusing on both, internal consistency structure and adequate factor correlations, we found that a six-factor solution fitted best to our data. Our results are in accordance with the theoretical assumption of the MDS Task Force [7] and recent work on the factorial structure of cognition in PD [8, 9] that differentiate between executive, long term memory, and retrieval ability as well as language and visual function. In contrast to others [8], we identified six instead of three domains. The rotation algorithm, the greater sample size, and our larger number of neuropsychological tasks may account for this difference. It is well known that the PD phenotype can vary largely. Thus, our more homogenous cohort without potential genetic variants of PD or other confounding factors could also have influenced the results.

In line with previous observations [8], verbal fluency performance was more closely related to psychomotor speed and language tasks than to other frontal lobe assessments (please compare Factor 1). Thus, it may be concluded that the verbal fluency task addresses a different cognitive aspect than the other frontal lobe instruments used here. This interpretation is further supported by the finding that semantic fluency impairment reflects structural grey matter changes in regions that are known to be involved in language networks [32]. Moreover, this task has been found to correlate with disease severity and motor assessment, which may explain an association to psychomotor speed performance in patients with PD [33, 34].

Another interesting finding is that the BAXT, an inventory for the assessment of ideomotor apraxia, was closely related to other frontal lobe tests in our PD cohort. Actually, patients with frontal lobe dysfunction may also show signs of ideomotor apraxia [35]. PD patients are known to suffer from an action-sequence planning deficit [36] that can at least partly explain the clinical signs of apraxia in PD. Recently, a strong association of finger dexterity with praxis function but not with the Parkinson's symptoms has been described [37]. This finding indicates that impaired finger dexterity in PD probably has an apraxic component, which is clinically more apparent in later disease stages. It thus seems that ideomotor deficits may rather contribute to an incorrect selection of action sequences than to a dysfunction in action semantics as suggested for patients with parietal lobe involvement. Indeed, symptoms of apraxia have been reported repeatedly in PD, although they are not as frequent and evident as in other neurodegenerative disorders [38, 39]. It was not the scope of this study to clarify which mechanism causes impairment in different cognitive domains or even in ideomotor apraxia. Nevertheless, our results suggest that apraxia could be a variant of the dysexecutive syndrome in PD. It might be interesting to address this hypothesis in future research.

The two logical memory subtests of the WMS-R did not reveal high loadings on Factor 2 (list learning and recall). In contrast to the CERAD memory tests, the verbal recall of the logical memory tasks may be more demanding with respect to working memory or metamemory, because it requires memory self-monitoring [40]. Thus, one may argue that the corresponding test performance is more dependent on frontal lobe functions [41]. Additionally, impaired logical memory abilities are known to be related to a decline in dopaminergic activity in the basal ganglia in both, healthy persons and PD patients [42–45]. This finding also supports the assumption that logical memory assessments address aspects of frontal lobe and working memory function and additionally may mirror dopaminergic dysfunction.

### 4.2. Cluster Analysis

#### 4.2.1. Cognitive Profile of Clusters

Based on the individual factor scores, both analyses revealed two independent groups with a subdivided, domain structure regarding the most affected cognitive functions. Crucially, the groups clustered even more closely without PDD patients (see Figure 1), arguing for a validation of the present grouping by our second analysis.

Cluster-II patients without dementia reported more ADL dysfunctions beyond their objectively more advanced cognitive decline. Interestingly, the CamPaIGN study showed that the PDD diagnosis at followup was linked to poorer semantic fluency at baseline and reduced visuoconstruction [4]. Others found that cognitive progression is strongly associated with memory and visuoconstructive skills [46, 47]. Likewise, all these functions were still more impaired in our overall more affected Cluster-II, even without PDD.

Currently, we can only speculate that Cluster-I and Cluster-II patients suffer from different pathologies. Dopaminergic loss modulates cognition (e.g., attention and psychomotor speed) especially in the early stages [48]. Interestingly, Cluster-I patients showed reduced attention as indicated by the corresponding factor scores. In contrast, advanced PD affects a broad range of cognitive abilities [49] as confirmed by the present Cluster-II. Since this cannot be fully attributed to dopaminergic loss [50], the extent of Lewy body pathology [51], an imbalance of other neurotransmitter systems, a primarily cholinergic deficit [52, 53], or Alzheimer's histopathology [54] should be considered.

#### 4.2.2. Implications for the Characterization of Cognitive Impairment in PD

At first sight there seems to be a contradiction between the cognitive profiles revealed by the two different analyses, that is, by either standard or factor scores. The comparison to the commonly used standard scores showed, as would be expected, that the Cluster-II patients are more impaired in almost all neuropsychological tests. The factor score analysis, however, identified a different cognitive profile in this formerly homogenous patient group (see Figure 1). Crucially, one needs to keep in mind that both, the standardized factor score and the standard score refer to the patients' individual test performance. The major difference is that the results are compared to different standardized group norms. The factor score represents the performance of one individual person in relation to the average performance of the total PD cohort. In contrast, the standard score specifies the individual test performance by normative data from healthy controls comparable with respect to age and education. Most important for the present study is that only the combination of these two sources revealed that patients of Cluster-I are predominately affected in attention performance, visual spatial abilities, and logical memory but not in the other cognitive domains. In contrast, patients of Cluster-II suffer additionally to the impairment of those of Cluster-I from a more extensive impairment in memory, frontal lobe function, fluency, and naming ability. Our alternative approach of analyzing the data driven standardized factor scores (instead of standardized percentile rank scores) offers the opportunity for a more precise differentiation within the PD group. Actually, the presence of two cognitive profiles within the group of PD patients without dementia could only be detected by the use of factor scores and not by the commonly applied standard scores.

At present, the PD-MCI concept [7, 55] is defined theoretically by the severity of dysfunction in one or more cognitive domains. However, its predictive value has not yet been proven [56]. One main difficulty is the heterogeneity of affected cognitive domains and severity of cognitive dysfunction in PD which is supported by many previous [1] as well as our present data. To date, it remains open which of the various cut-off values are most predictive of PDD and which neuropsychological tasks might be the most promising to identify PD patients at risk for dementia according to the MCI concept.

Following our preliminary results of a clinical sample, we argue that a global neuropsychological (domain) score based on standardized assessments and compared to population based PD norms (e.g., factor scores) may help reflect the level of cognitive impairment and its progression more appropriately than various or even single cut-off scores from healthy control subjects. Such PD norms may offer the possibility to specify for each single PD patient whether the deficits occur to a greater or lesser extent compared to other PD patients, resulting in a more sensitive characterization of both kind and severity of cognitive impairment. Such PD norms should be derived from representative PD samples that undergo a well-defined, standardized neuropsychological test battery that is widely used and accepted, for example, following MDS Task Force recommendations [7].

## 5. Limitations

It needs to be considered that the generalizability of our results is limited by the small sample size. Still, although the cohort is not population based, our PDD patients present the well-known phenotype, that is, they were older and had a longer disease duration (please see [2, 57]).

Further, we are aware of the methodological limitations of explorative factor analyses. Nevertheless, our data driven approach provides a useful alternative to generate even more specific hypotheses on the resulting factor structure, which have to be verified by future research using, for example, confirmatory models. Such studies may offer a promising perspective to evaluate PD patients' cognitive progression or conversion to dementia over time.

## 6. Conclusions

Our data-driven approach suggests at least two different subtypes of cognitive impairment in PD, which are rather independent of motor function, disease duration, and PD medication but do have an impact on activities of daily living. Moreover, our data driven approach confirms the cognitive domains suggested by the consensus guidelines.

## Supplementary Material

Supplementary Table 1: Results of the exploratory factor analysis and consistency analysis (Cronbach's alpha coefficients) of a five-factor model of cognition in Parkinson's Diesease patients indicating highest internal consistency for the presented six-factor solution.

## Figures and Tables

**Table 1:** Demographic, clinical, and neuropsychological characteristics of the two PD groups (PDD patients included) as identified by the first hierarchical cluster analysis.

	Total group of PD and PDD	Cluster-I PD only*	Cluster-II PD and PDD*	*P* value
Number, (%)	121 (100.0)	50 (41.3)	71 (58.7)	
Male gender, *n* (%)	81 (66.9)	33 (66.0)	48 (67.6)	0.85
Age at evaluation, years	68.7 ± 6.9	66.1 ± 6.7	70.6 ± 6.4	<0.001
Neurological assessment				
Disease duration, years	6.6 ± 5.1	5.8 ± 4.7	7.1 ± 5.2	0.17
UPDRS-III motor score (on state)	28.3 ± 11.5	25.3 ± 11.7	30.5 ± 11.0	0.01
Hoehn and Yahr stage, *n* (%)				
1	12 (9.9)	6 (12.0)	6 (8.5)	
1.5	5 (4.1)	3 (6.0)	2 (2.8)	
2	49 (40.5)	24 (48.0)	25 (35.2)	0.06
2.5	32 (26.5)	14 (28.0)	18 (25.4)	
3	16 (13.2)	3 (6.0)	13 (18.3)	
4	7 (5.8)	0 (0)	7 (9.9)	
Medication, daily dose				
Levodopa dose (mg)	351.4 ± 304.7	330.3 ± 343.6	366.3 ± 275.6	0.55
Levodopa equivalent dose (mg)	573.8 ± 417.2	585.0 ± 470.2	566.0 ± 378.8	0.35
Antidepressants, *n* (%)	28 (23.1)	9 (18.0)	19 (26.8)	0.12
Neuroleptics, *n* (%)	14 (11.6)	1 (2.0)	13 (18.3)	0.11
PD patients with dementia, PDD	24 (19.8)	0 (0)	24 (33.8)	<0.001
MMSE (raw score)	26.6 ± 2.6	28.1 ± 1.5	25.5 ± 2.6	<0.001
Beck's Depression inventory	8.7 ± 5.7	7.1 ± 4.7	9.9 ± 6.0	0.009
Neuropsychiatric inventory	4.7 ± 7.3	3.5 ± 5.5	5.5 ± 8.2	0.22
Parkinson's disease Questionnaire-PDQ-39	5.4 ± 4.2	3.4 ± 3.1	6.8 ± 4.3	0.001
NAI: NAA-ADL inventory, patients' self-assessment	48.8 ± 32.4	67.2 ± 22.0	35.9 ± 32.4	<0.001
NAI: NAB-ADL inventory, caregivers' assessment	50.3 ± 31.0	67.1 ± 25.1	38.4 ± 29.4	<0.001

Factor scores	Standardized values of the total PD cohort (PD norms)	Mean group performance in relation to the standardized values, that is, below (−) versus above (+) the average of the total PD cohort		

Factor 1, frontal lobe function	0 ± 1	0.41 ± 0.71	−0.29 ± 1.07	0.005
Factor 2, word-list memory and recall	0 ± 1	0.56 ± 0.90	−0.39 ± 0.87	<0.001
Factor 3, attention	0 ± 1	−0.58 ± 0.87	0.37 ± 0.91	<0.001
Factor 4, logical memory	0 ± 1	−0.68 ± 1.06	0.48 ± 0.60	<0.001
Factor 5, praxis and visual perception	0 ± 1	−0.92 ± 0.90	0.56 ± 0.76	<0.001
Factor 6, fluency and naming ability	0 ± 1	0.62 ± 0.84	−0.44 ± 0.86	<0.001

Neuropsychological assessment	Mean group performance in relation to the standardized values provided by the test manuals, that is, below (−) and above (+) the average of healthy control subjects			

Factor 1:				
Trail Making Test, part B	49.8 ± 39.3	75.2 ± 29.8	32.0 ± 35.3	<0.001
Tower of London	39.0 ± 26.7	48.5 ± 24.0	32.3 ± 26.6	0.024
NAI: digit span	56.3 ± 31.4	70.3 ± 28.7	46.5 ± 29.6	<0.001
NAI: figure test	52.1 ± 27.2	62.6 ± 20.1	44.7 ± 29.1	0.006
Berlin Apraxia Test (raw score)	35.7 ± 5.5	38.7 ± 3.2	33.7 ± 5.9	<0.001
Factor 2:				
CERAD: word-list memory	29.9 ± 27.3	48.5 ± 16.8	16.8 ± 20.3	<0.001
CERAD: word-list recall	36.9 ± 30.0	54.8 ± 29.1	24.2 ± 23.7	<0.001
CERAD: word-list recognition	40.7 ± 34.5	57.2 ± 30.3	20.0 ± 32.7	<0.001
CERAD: word-list intrusion	42.3 ± 33.7	53.7 ± 28.0	34.2 ± 34.0	0.005
Factor 3:				
TAP: phasic alertness	55.4 ± 29.2	44.2 ± 25.7	63.3 ± 29.1	0.001
TAP: Go-Nogo, median RT	40.5 ± 33.5	60.6 ± 29.1	26.4 ± 29.0	<0.001
Factor 4:				
WMS-R: logical memory I	24.4 ± 26.2	41.9 ± 26.9	12.0 ± 17.0	<0.001
WMS-R: logical memory II	25.9 ± 26.2	45.0 ± 26.1	12.5 ± 16.0	<0.001
Factor 5:				
CERAD: praxis	40.2 ± 35.7	63.6 ± 29.9	23.7 ± 29.8	<0.001
CERAD: praxis delay	35.6 ± 35.8	59.0 ± 34.8	19.2 ± 26.2	<0.001
VOSP: object decision	42.4 ± 30.1	56.4 ± 29.6	32.5 ± 26.5	0.001
Factor 6:				
CERAD: verbal fluency	30.0 ± 28.1	52.8 ± 27.7	24.2 ± 21.8	<0.001
CERAD: Boston naming test	46.9 ± 33.2	63.1 ± 28.1	35.4 ± 31.9	<0.001
Trail Making Test, part A	45.8 ± 35.4	70.2 ± 28.0	28.6 ± 29.6	<0.001

Data are given as mean ± SD; lower standard (that is, percentile rank) scores in neuropsychological tests indicate poorer performance except for the MMSE; UPDRS-III: Unified Parkinson's Disease Rating Scale part III; *P* values are corrected for age and UPDRS-III motor score; %: Percentage; PD: Parkinson's disease; PDD: Parkinson's disease with dementia; LEDD: levodopa equivalence daily dose according to the following conversion rates: 100 mg Levodopa equalling 125 mg Levodopa sustained release, 1 mg Pergolide, 1 mg Pramipexol, 5 mg Ropinirole, 5 mg Rotigotin, 10 mg Bromocriptine, 10 mg Apomorphine, 1/5 Entacapone, 1.5 mg Cabergoline. Additionally, 5% was added to the total Levodopa dose for every 5 mg of Selegiline or 1 mg of Rasagiline, up to a maximum of 10%; MMSE: Minimental State Examination; NAI: Nuernberger Alters Inventar; RT: reaction time; *Grouping of patients with PDD following the first hierarchical cluster analysis.

**Table 2:** Demographic, clinical, and neuropsychological characteristics of the two PD groups (PDD patients excluded) as identified by the second hierarchical cluster analysis.

	PDD only	Cluster-I PD only	Cluster-II PD only	*P* value
Number, (%)	24 (19.8)	43 (35.6)	54 (44.6)	
Male gender, *n* (%)	18 (75.0)	28 (65.1)	35 (64.8)	0.97
Age at evaluation, years	74.2 ± 5.9	65.7 ± 6.0	68.7 ± 6.5	0.02
Neurological assessment				
Disease duration, years	9.5 ± 5.6	5.6 ± 4.3	6.1 ± 4.9	0.66
UPDRS-III motor score (on state)	37.5 ± 11.3	25.3 ± 11.5	26.7 ± 9.6	0.52
Hoehn and Yahr stage, *n* (%)				
1	0 (0)	6 (14.0)	6 (11.1)	
1.5	0 (0)	3 (7.0)	2 (3.7)	
2	7 (29.2)	21 (48.8)	21 (38.9)	0.60
2.5	3 (12.5)	11 (25.6)	18 (33.3)	
3	8 (33.3)	2 (4.7)	6 (11.1)	
4	6 (25.0)	0 (0)	1 (1.9)	
Medication, daily dose				
Levodopa dose (mg)	457.2 ± 256.0	323.5 ± 352.6	326.6 ± 277.2	0.57
Levodopa equivalent dose (mg)	665.7 ± 407.8	554.7 ± 435.7	548.2 ± 408.3	0.82
Antidepressants, *n* (%)	7 (29.2)	8 (18.6)	13 (24.1)	0.14
Neuroleptics, *n* (%)	7 (29.2)	1 (2.3)	6 (11.1)	0.67
MMSE (raw score)	23.0 ± 2.7	28.1 ± 1.5	26.9 ± 1.5	0.003
Beck's Depression Inventory	11.6 ± 6.2	7.1 ± 4.8	8.8 ± 5.8	0.06
Neuropsychiatric inventory	9.5 ± 10.3	3.3 ± 5.1	3.3 ± 5.1	0.73
Parkinson's disease questionnaire-PDQ-39	10.4 ± 4.2	3.5 ± 3.1	4.9 ± 3.2	0.03
NAI: NAA-ADL inventory, patients' self-assessment	8.8 ± 16.9	67.6 ± 22.3	51.7 ± 28.9	0.002
NAI: NAB-ADL inventory, caregivers' assessment	11.5 ± 10.3	66.8 ± 24.6	54.4 ± 27.1	0.03

Factor scores	Mean group performance in relation to the standardized values (0 ± 1), that is, below (−) and above (+) the average of the total PD cohort			

Factor 1, frontal lobe function	−1.11 ± 0.86	0.32 ± 0.71	0.23 ± 0.92	0.64
Factor 2, word-list memory and recall	−0.76 ± 0.90	0.73 ± 0.84	−0.25 ± 0.77	<0.001
Factor 3, attention	0.61 ± 0.98	−0.56 ± 0.92	0.17 ± 0.85	<0.001
Factor 4, logical memory	0.51 ± 0.67	−0.73 ± 1.13	0.35 ± 0.62	<0.001
Factor 5, praxis and visual perception	0.92 ± 0.70	−0.67 ± 0.90	0.14 ± 0.79	<0.001
Factor 6, fluency and naming ability	−0.86 ± 0.81	0.69 ± 0.81	−0.17 ± 0.84	<0.001

Neuropsychological assessment	Mean group performance in relation to the standardized values provided by the test manuals, that is, below (−) and above (+) the average of healthy control subjects			

Factor 1:				
Trail Making Test, part B	5.3 ± 15.1	75.9 ± 30.2	48.9 ± 35.0	0.001
Tower of London	14.8 ± 22.0	47.7 ± 25.1	42.9 ± 23.8	0.46
NAI: digit span	39.0 ± 32.2	66.9 ± 29.5	55.6 ± 29.4	0.04
NAI: figure test	26.2 ± 29.4	62.4 ± 21.3	55.3 ± 23.0	0.12
Berlin Apraxia Test (raw score)	29.3 ± 6.6	38.6 ± 3.3	36.4 ± 3.9	0.005
Factor 2:				
CERAD: word-list memory	12.7 ± 21.7	51.2 ± 25.6	21.9 ± 19.4	<0.001
CERAD: word-list recall	18.0 ± 24.5	60.2 ± 27.4	26.7 ± 21.8	<0.001
CERAD: word-list recognition	15.5 ± 24.0	62.8 ± 27.8	34.2 ± 33.3	<0.001
CERAD: word-list intrusion	20.8 ± 31.9	56.9 ± 29.0	40.1 ± 32.8	0.008
Factor 3:				
TAP: phasic alertness	68.7 ± 32.9	43.6 ± 27.1	59.0 ± 25.9	0.007
TAP: Go-Nogo, median RT	16.5 ± 28.1	60.0 ± 30.1	37.3 ± 30.4	<0.001
Factor 4:				
WMS-R: logical memory I	8.5 ± 16.5	43.2 ± 27.9	16.4 ± 18.8	<0.001
WMS-R: logical memory II	7.5 ± 12.2	47.3 ± 27.2	17.1 ± 17.2	<0.001
Factor 5:				
CERAD: praxis	13.1 ± 27.4	63.6 ± 31.3	33.7 ± 31.1	<0.001
CERAD: praxis delay	10.3 ± 21.5	58.9 ± 35.2	28.3 ± 30.5	<0.001
VOSP: object decision	19.9 ± 19.6	56.6 ± 29.2	41.1 ± 28.6	0.04
Factor 6:				
CERAD: verbal fluency	17.6 ± 20.8	55.4 ± 27.3	28.7 ± 22.3	<0.001
CERAD: Boston naming test	21.3 ± 28.8	64.4 ± 28.3	44.3 ± 30.8	0.001
Trail Making Test, part A	9.0 ± 16.8	71.8 ± 27.3	41.4 ± 30.6	<0.001

Data are given as mean ± SD; lower standard (i.e. percentile rank) scores in neuropsychological tests indicate poorer performance except for the MMSE; UPDRS-III: Unified Parkinson's Disease Rating Scale part III; *P* values are corrected for age; %: Percentage; PD: Parkinson's disease; PDD: Parkinson's disease with dementia; LEDD: Levodopa equivalence daily dose according to the following conversion rates: 100 mg Levodopa equalling 125 mg Levodopa sustained release, 1 mg Pergolide, 1 mg Pramipexol, 5 mg Ropinirole, 5 mg Rotigotin, 10 mg Bromocriptine, 10 mg Apomorphine, 1/5 Entacapone, 1.5 mg Cabergoline. Additionally, 5% was added to the total levodopa dose for every 5 mg of Selegiline or 1 mg of Rasagiline, up to a maximum of 10%; MMSE: Minimental State Examination; NAI: Nuernberger Alters Inventar; RT: reaction time.

**Table 3:** Results of the exploratory factor analysis and the consistency analysis on the neuropsychological test results of all 121 patients indicating a six-factor model of cognition in PD.

Factor interpretation	Factor 1	Factor 2	Factor 3	Factor 4	Factor 5	Factor 6
	frontal lobe function	word-list memory and recall	attention	logical memory	praxis and visual perception	fluency and naming ability
Tower of London	0.62					
Trail Making Test, part B	0.64					
NAI: digit span	0.65					
NAI: figure test	0.69					
Berlin Apraxia Test (raw score)	0.66					
CERAD: word-list memory		0.77				
CERAD: word-list recall		0.84				
CERAD: word-list recognition		0.71				
CERAD: word-list intrusion		0.77				
TAP: phasic alertness			−0.80			
TAP: Go-Nogo, median RT			0.70			
WMS-R: logical memory I				0.88		
WMS-R: logical memory II				0.87		
CERAD: praxis					0.83	
CERAD: praxis delay					0.83	
VOSP: object decision					0.62	
CERAD: verbal fluency						0.84
CERAD: Boston naming test						0.77
Trail Making Test, part A						0.66

Variance explained (%)	33.51	8.15	6.78	5.90	5.70	5.19
Cronbach's alpha coefficient	0.67	0.78	−0.85	0.86	0.70	0.70

Analyses are based on standard norms (i.e percentile rank scores, PR: indicating the patient's relative position in the norm group with a range between 0 and 100) of healthy German control subjects as published in the manuals; data are corrected either for age (NAI, WMS-R, VOSP) or for age and education (CERAD, TAP, TMT, TL-D). Only for the BAXT raw data were used; CERAD: Consortium to Establish a Registry For Alzheimer's Disease, German version; WMS-R: Wechsler Memory Scale-Revised; NAI: Nuernberger Alters Inventory; VOSP: Visual Object and Space Perception battery; TAP: Test of Attentional Performance; RT: Reaction Time.
