# Supplementary material for: Cognitive Profiles in Parkinson's Disease and Their Relation to Dementia: A Data-Driven Approach
Source: Int J Alzheimers Dis. 2012 Oct 18;2012:910757. doi: 10.1155/2012/910757 (PMC3483831; doi:10.1155/2012/910757)
Supplement: Supplementary file 1 — Supplementary Table 1: Results of the exploratory factor analysis and consistency analysis (Cronbach's alpha coefficients) of a five-factor model of cognition in Parkinson's Diesease patients indicating highest internal consistency for the presented six-factor solution. [file 910757.f1.docx]

**Supplemented Table 1.** Results of the exploratory factor analysis and consistency analysis (Cronbach’s alpha coefficients) of a five-factor model of cognition in Parkinson patients indicating highest internal consistency for the presented six-factor solution.

|  | Factor 1 | Factor 2 | Factor 3 | Factor 4 | Factor 5 |
| --- | --- | --- | --- | --- | --- |
| *Tower of London* | 0.62 |  |  |  |  |
| *Trail Making Test*, Part B | 0.72 |  |  |  |  |
| *NAI:* Digit Span | 0.69 |  |  |  |  |
| *NAI:* Figure Test | 0.66 |  |  |  |  |
| *Berlin Apraxia Test* (raw score) | 0.73 |  |  |  |  |
| *CERAD:* Word-list memory |  | 0.78 |  |  |  |
| *CERAD:* Word-list recall |  | 0.84 |  |  |  |
| *CERAD:* Word-list recognition |  | 0.78 |  |  |  |
| *CERAD:* Word-list intrusion |  | 0.70 |  |  |  |
| *CERAD:* Verbal fluency |  |  | 0.48 |  |  |
| *Trail Making Test,* Part A |  |  | 0.61 |  |  |
| *TAP:* Phasic Alertness |  |  | -0.75 |  |  |
| *TAP:* Go-Nogo, Median RT |  |  | 0.65 |  |  |
| *WMS-R:* Logical Memory I |  |  |  | 0.87 |  |
| *WMS-R:* Logical Memory II |  |  |  | 0.86 |  |
| *CERAD:* Boston Naming Test |  |  |  |  | 0.64 |
| *CERAD:* Praxis |  |  |  |  | 0.77 |
| *CERAD:* Praxis-Delay |  |  |  |  | 0.76 |
| *VOSP:* Object decision |  |  |  |  | 0.64 |
| Variance explained (%) | 33.51 | 8.15 | 6.78 | 5.90 | 5.70 |
| Cronbachs alpha coefficient | 0.67 | 0.78 | 0.31 | 0.86 | 0.69 |

Analyses are based on standard norms (i.e. percentile rank scores, PR, indicating the patient’s relative position in the norm group with a range between 0 and 100) of healthy German control subjects as published in the manuals; Data are corrected either for age (NAI, WMS-R, VOSP) or for age and education (CERAD, TAP, TMT, TL-D). Only for the BAXT raw data were used; CERAD. Consortium for the Registry for Alzheimer's Disease, German version; WMS-R. Wechsler Memory Scale-Revised; NAI. Nuernberger Alters Inventory; VOSP. Visual Object and Space Perception Battery; TAP. Testbatterie zur Aufmerksamkeitspruefung; RT. Reaction time.
